# Supplementary material for: Disease progression model anchored around clinical diagnosis in longitudinal cohorts: example of Alzheimer’s disease and related dementia
Source: BMC Med Res Methodol. 2023 Sep 5;23:199. doi: 10.1186/s12874-023-02009-0 (PMC10478286; doi:10.1186/s12874-023-02009-0)
Supplement: Supplementary file 1 — Additional file 1. [file 12874_2023_2009_MOESM1_ESM.pdf]

Supplementary Material for: Disease progression model anchored around clinical diagnosis in longitudinal cohorts: example of Alzheimer's disease and related dementia

Jérémie Lespinasse[1,2,3,*], Carole Dufouil[1,2,3,†,*], and Cécile Proust-Lima[1,2,†,*]

[1] *Univ. Bordeaux, Inserm, Bordeaux Population Health Research Center, BPH, U1219, F-33000 Bordeaux, France*

[2] *Inserm, CIC1401-EC, F-33000 Bordeaux, France*

[3] *Pole de santé publique, Centre Hospitalier Universitaire (CHU) de Bordeaux, F-33000 Bordeaux, France*

[†] *co-last authors*

[*] *Correspondence : cecile.proust-lima@inserm.fr*

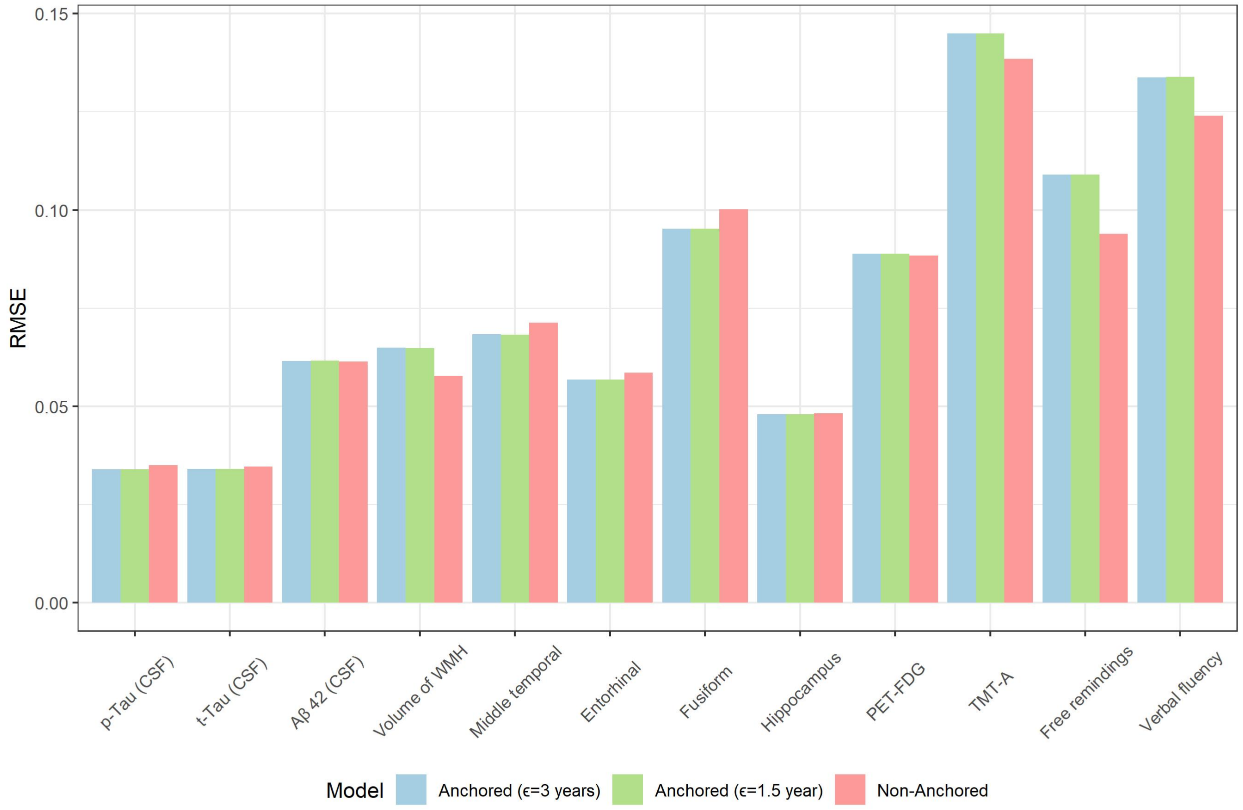

Figure S1 – Residual mean square error per marker for the main disease progression anchored model (DPAM) with constraint of 1.5 years around the observed clinical diagnosis, the DPAM with a weaker constraint of 3 years, and a non-anchored disease progression model, the MEMENTO Cohort, France, 2011-2019 (N=2186).

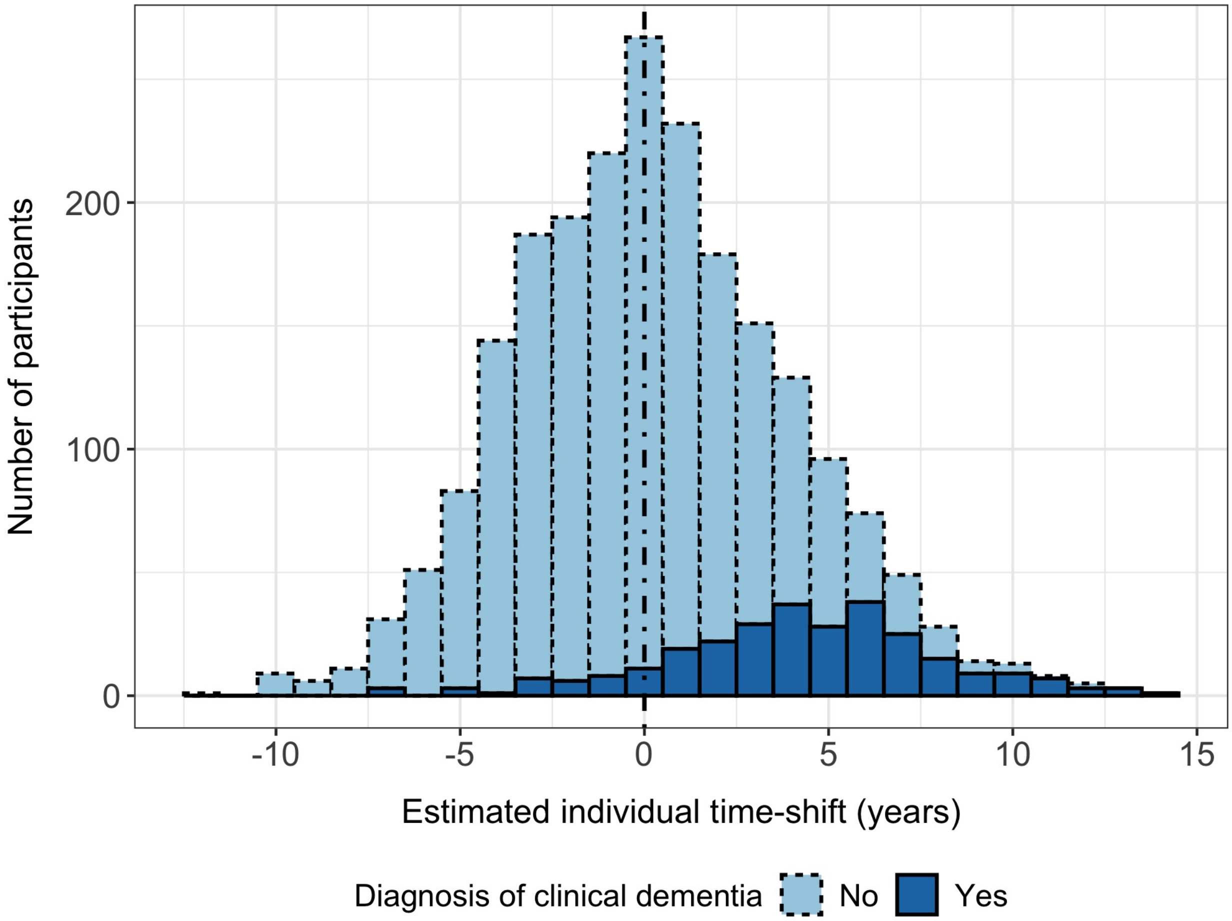

Figure S2 – Posterior distribution of the estimated individual time shifts from a non-anchored disease progression model, the MEMENTO Cohort, France, 2011-2019 (N=2186).

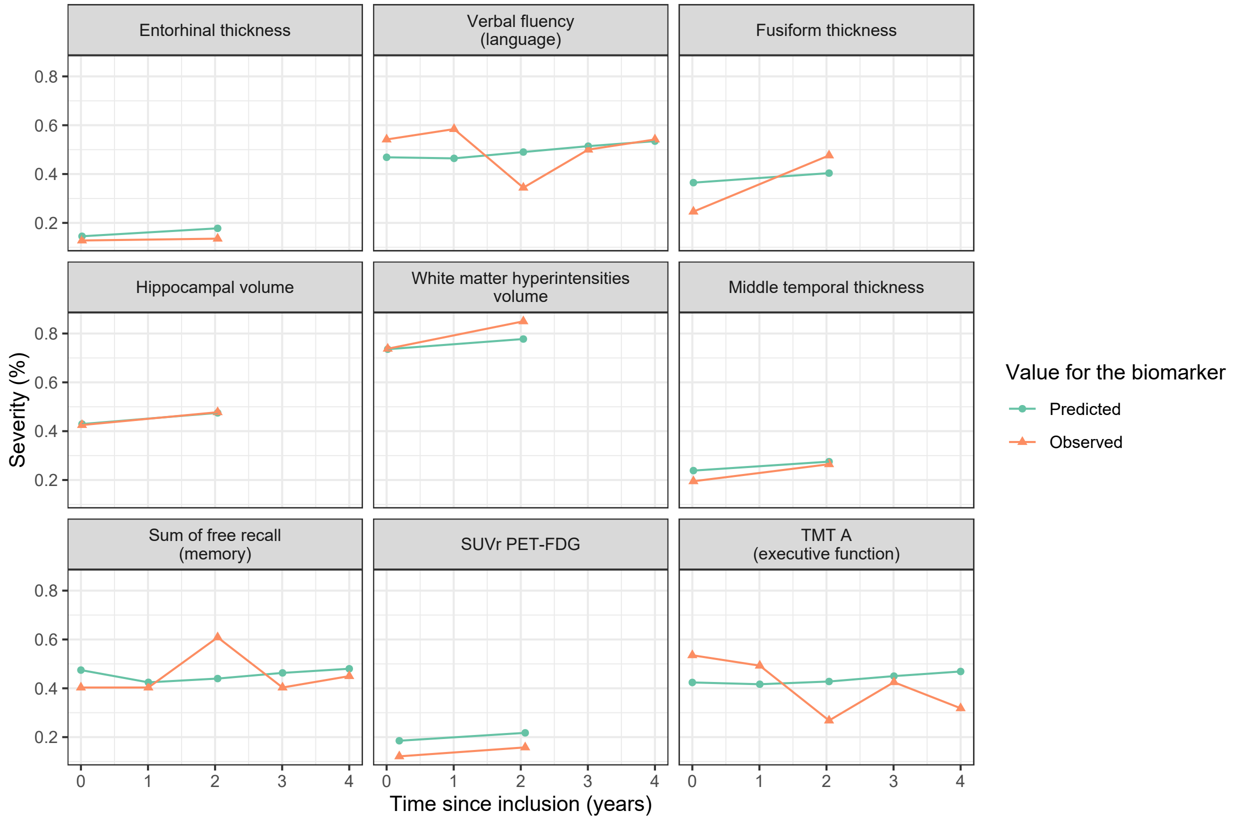

Figure S3 – Observations and individual predictions from the anchored disease progression model reported in the percentile scale according to time since enrollment for participant # 1 randomly selected from the analytical sample, the MEMENTO Cohort, France, 2011-2019 (N=2186).

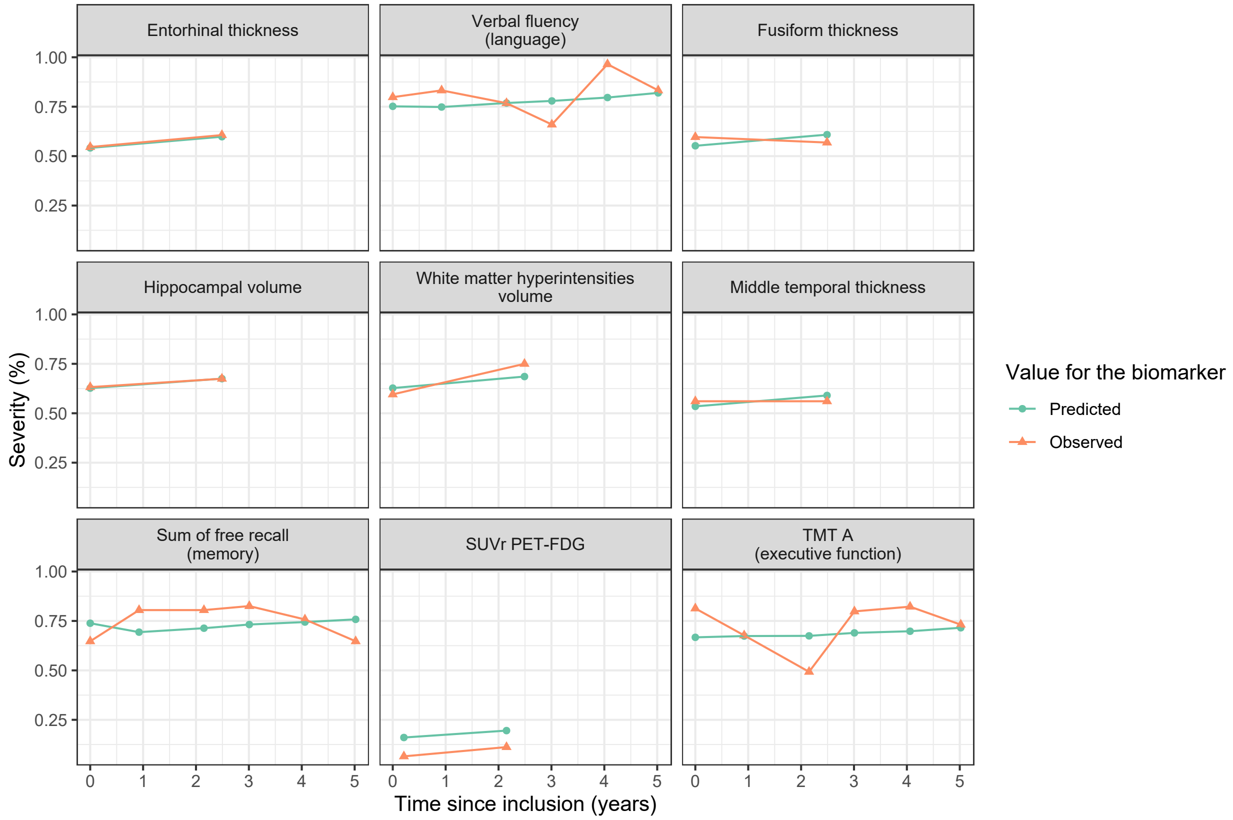

FIGURE S4 – Observations and individual predictions from the anchored disease progression model reported in the percentile scale according to time since enrollment for participant # 2 randomly selected from the analytical sample, the MEMENTO Cohort, France, 2011-2019 (N=2186).

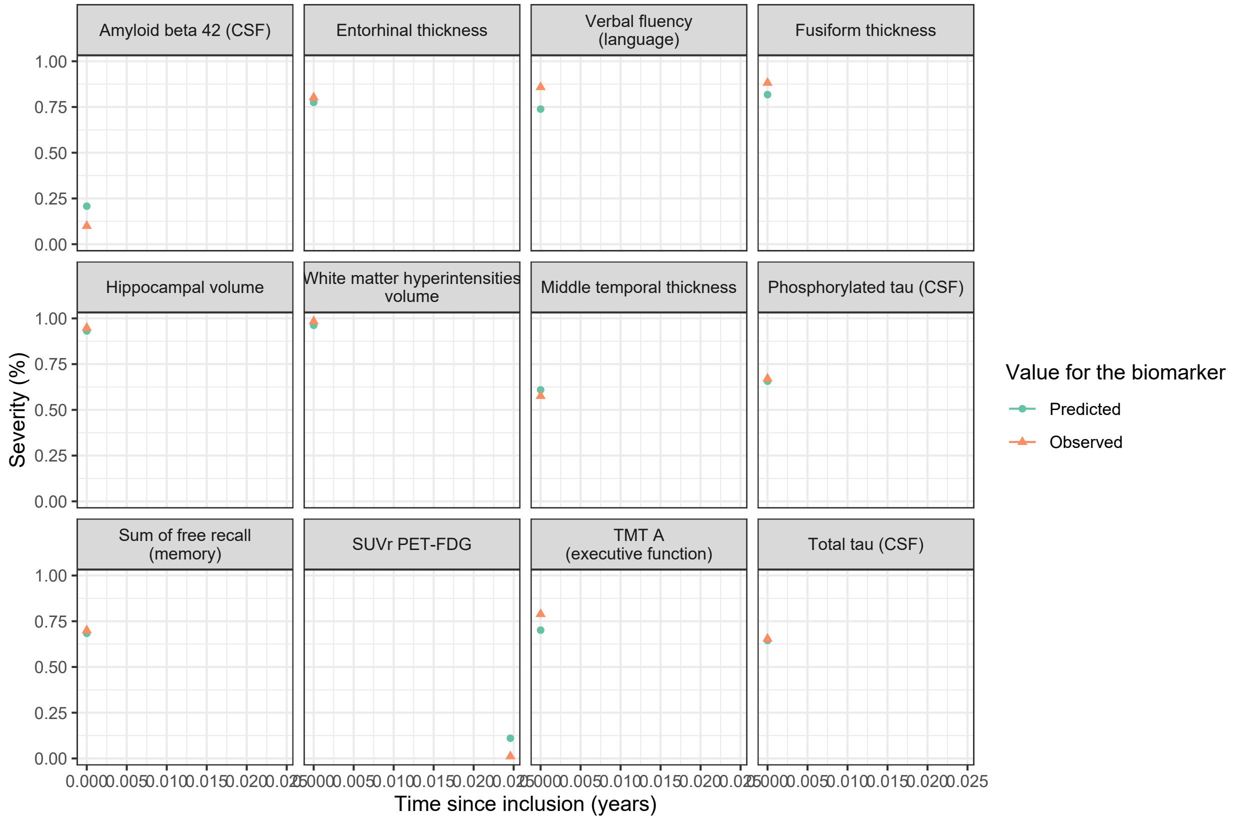

FIGURE S5 – Observations and individual predictions from the anchored disease progression model reported in the percentile scale according to time since enrollment for participant # 3 randomly selected from the analytical sample, the MEMENTO Cohort, France, 2011-2019 (N=2186).

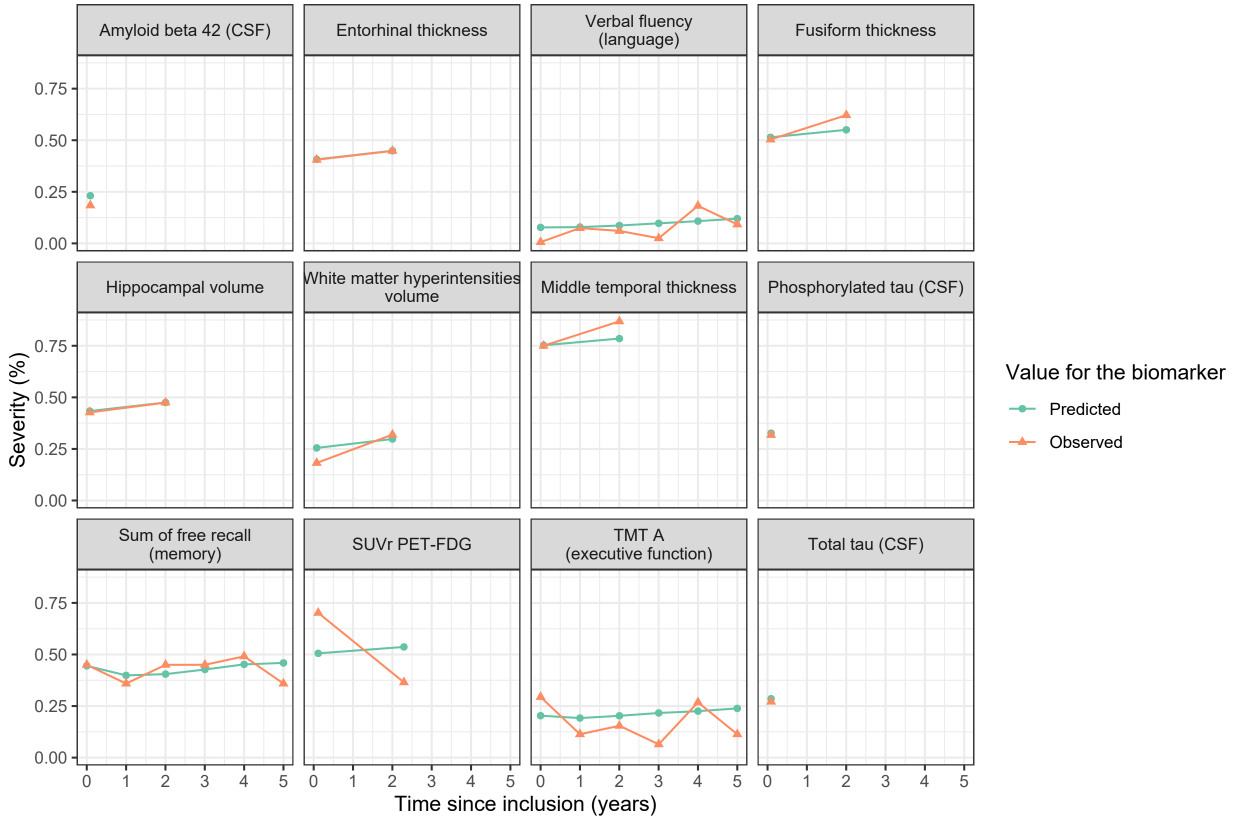

FIGURE S6 – Observations and individual predictions from the anchored disease progression model reported in the percentile scale according to time since enrollment for participant # 4 randomly selected from the analytical sample, the MEMENTO Cohort, France, 2011-2019 (N=2186).

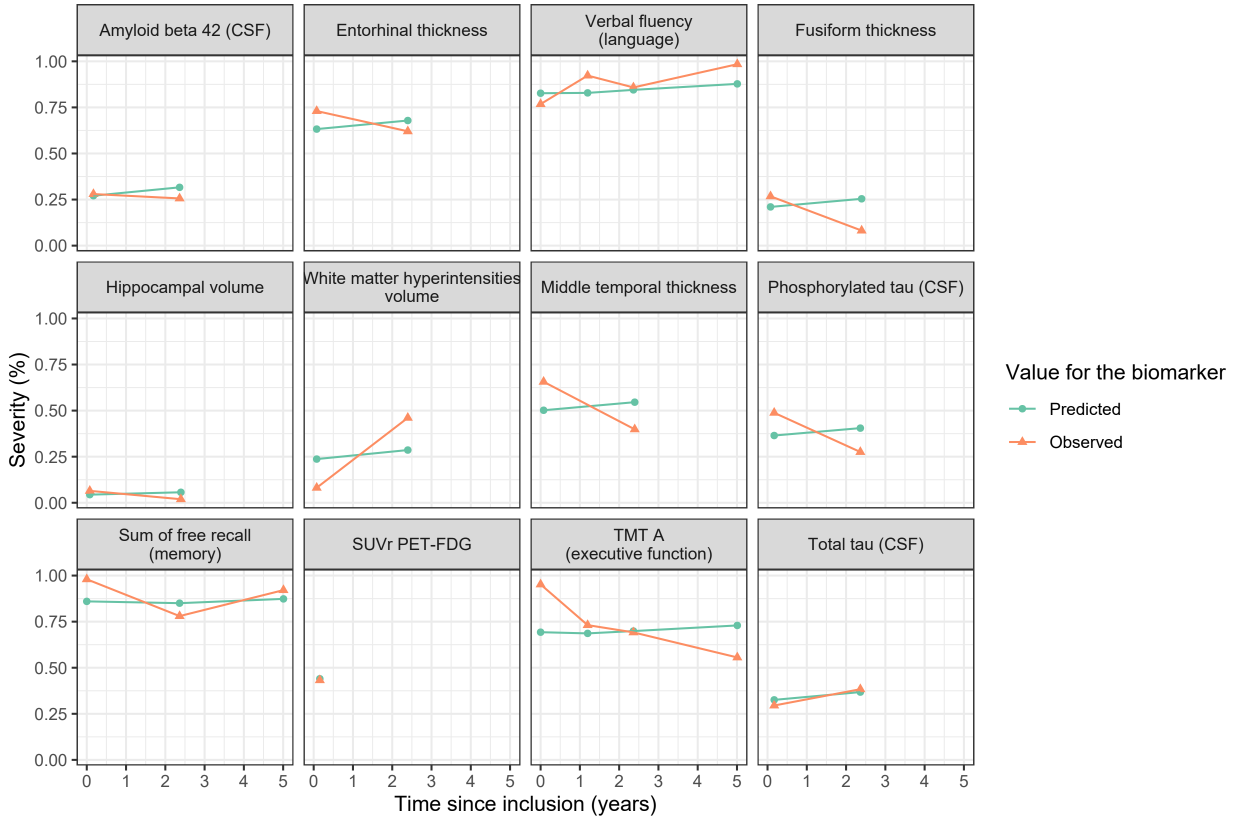

FIGURE S7 – Observations and individual predictions from the anchored disease progression model reported in the percentile scale according to time since enrollment for participant # 5 randomly selected from the analytical sample, the MEMENTO Cohort, France, 2011-2019 (N=2186).

TABLE S1 : List of the Memento Study Group members

Name	Degree	Location	Role
Michèle Allard	MD, PhD	Memory Resource and Research Centre of Bordeaux, CHU de Bordeaux, Hôpital Xavier Arnozan, F-33000, Bordeaux, France	Co-investigator
Sandrine Andrieu	MD, PhD	Memory Resource and Research Centre of Toulouse, CHU de Toulouse, Hôpital La Grave-Casselardit, F-31000, Toulouse, France	Co-investigator
Pierre Anthony	MD, PhD	Memory Resource and Research Centre of Colmar, Hôpitaux Civils de Colmar, F-68000, Colmar, France	Co-investigator
Christine Astier	MD	Memory Resource and Research Centre of Strasbourg, Hôpitaux Universitaires de Strasbourg, F-67000, Strasbourg, France	Co-investigator
Alexandre Augier	MD, PhD	Memory Clinic, Hôpital Avicenne, AP-HP, Hôpitaux Universitaires Paris-Seine-Saint-Denis, F-93009, Bobigny, France	Co-investigator
Nicolas Auguste	MD	Memory Resource and Research Centre of Saint-Etienne, CHU de Saint-Etienne, Hôpital de la Charité, F-42000, Saint-Etienne, France	Co-investigator
Sophie Auriacombe	MD, PhD	Memory Resource and Research Centre of Bordeaux, CHU de Bordeaux, Hôpital Pellegrin, F-33000, Bordeaux, France	Co-investigator
John Avet	MD, PhD	Memory Resource and Research Centre of Saint-Etienne, CHU de Saint-Etienne, Hôpital Nord, F-42000, Saint-Etienne, France	Co-investigator
Olivier Bailon	MD, PhD	Memory Clinic, Hôpital Avicenne, AP-HP, Hôpitaux Universitaires Paris-Seine-Saint-Denis, F-93009, Bobigny, France	Co-investigator
Anna-Chloé Balageas	MD	Memory Resource and Research Centre of Center Region, CHRU de Tours, Hôpital Bretonneau, F-37000, Tours, France	Co-investigator
Fabrice-Guy Barral	MD	Memory Resource and Research Centre of Saint-Etienne, CHU de Saint-Etienne, Hôpital Nord, F-42000, Saint-Etienne, France	Co-investigator
Jean Barré	MD	Memory Resource and Research Centre of Angers, CHU d'Angers, F-49000, Angers	Co-investigator
Annick Barthelaix	MD, PhD	Memory Resource and Research Centre of Angers, CHU d'Angers, F-49000, Angers	Co-investigator
Catherine Bayle	MD	Memory Resource and Research Centre of Paris Broca, AP-HP, Paris, France	Co-investigator
Olivier Beauchet	MD, PhD	Memory Resource and Research Centre of Angers, CHU d'Angers, F-49000, Angers	Co-investigator
Catherine Belin	MD, PhD	Memory Clinic, Hôpital Avicenne, AP-HP, Hôpitaux Universitaires Paris-Seine-Saint-Denis, F-93009, Bobigny, France	Co-investigator
Samia Belkacem	MD	Institute of Memory and Alzheimer's Disease (IM2A), Centre for NeuroImaging Research (CENIR), Brain and Spine Institute (ICM), UMR S 1127, Department of Neurology, AP-HP, Pitié-Salpêtrière University Hospital, Sorbonne Universities, Pierre et Marie Curie University, Paris, France	Co-investigator
Douraied Ben Salem	MD, PhD	Memory Resource and Research Centre of Brest, CHRU de Brest, F-29000, Brest, France	Co-investigator
Karim Bennys	MD	Memory Resource and Research Centre of Montpellier, CHU de Montpellier, Hôpital Gui de Chauliac, F-34000, Montpellier, France	Co-investigator
Géraldine Bera	MD	Laboratoire d'Imagerie Biomédicale, Sorbonne Universités, UPMC Univ Paris 06, Inserm U1146, CNRS UMR 7371, France NeuroSpin, I2BM, Commissariat à l'Energie Atomique, Paris, France	Co-investigator
Eric Berger	MD	Memory Resource and Research Centre of Besançon, CHU de Besançon, Hôpital Jean Minjoz, Hôpital Saint-Jacques, F-25000, Besançon, France	Co-investigator
Marc G Berger	MD, PhD	Memory Resource and Research Centre of Clermont-Ferrand, CHU de Clermont-Ferrand, F-63000, Clermont-Ferrand, France	Co-investigator
Emilie Bergouin	MD	Memory Resource and Research Centre of Dijon, CHU Dijon Bourgogne, Hôpital du Bocage, Hôpital de Champmaillot, F-21000, Dijon, France	Co-investigator
François Bertin-Hugault	MD	Memory Resource and Research Centre of Lyon, Hospices Civils de Lyon, Hôpital des Charpennes, F-69000, Lyon, France	Co-investigator
Guillaume Bertrand	MD	Memory Clinic, Hôpital Avicenne, AP-HP, Hôpitaux Universitaires Paris-Seine-Saint-Denis, F-93009, Bobigny, France	Co-investigator
François-Xavier Bertrand	MD, PhD	Memory Resource and Research Centre of Nantes, CHU de Nantes, F-44000, Nantes, France	Co-investigator
Catherine Beze	MD	Memory Resource and Research Centre of Center Region, CHRU de Tours, Hôpital Bretonneau, F-37000, Tours, France	Co-investigator
Valérie Boilet		Coordinating Centre, Inserm CIC-1401 Clinical Epidemiology, CHU de Bordeaux, F-33000, Bordeaux, France	Co-investigator
Stéphanie Bombois	MD, PhD	Institute of Memory and Alzheimer's Disease (IM2A), Brain and Spine Institute (ICM), UMR S 1127, Department of Neurology, AP-HP, Pitié-Salpêtrière University Hospital, Sorbonne Universities, Pierre et Marie Curie University, Paris, France	Co-investigator
Alain Bonafé	MD, PhD	Memory Resource and Research Centre of Montpellier, CHU de Montpellier, Montpellier, France	Co-investigator
Yasmina Boudali	MD	Memory Resource and Research Centre of Paris Broca, AP-HP, Paris, France	Co-investigator
Hatem Bouhladour	MD, PhD	Memory Resource and Research Centre of Besançon, CHU de Besançon, Hôpital Jean Minjoz, Hôpital Saint-Jacques, F-25000, Besançon, France	Co-investigator
Clémence	MD	Memory Resource and Research Centre of Paris Broca, AP-HP, Paris, France	Co-investigator

Boully			
Isabelle Bourdel-Marchasson	MD, PhD	Memory Resource and Research Centre of Bordeaux, CHU de Bordeaux, Hôpital Xavier Arnozan, F-33000, Bordeaux, France	Co-investigator
Vincent Bouteloup	PharmD	Coordinating Centre, Inserm CIC-1401 Clinical Epidemiology, CHU de Bordeaux, F-33000, Bordeaux, France	Co-investigator
Claire Boutet	MD	Institute of Memory and Alzheimer's Disease (IM2A), Centre for NeuroImaging Research (CENIR), Brain and Spine Institute (ICM), UMR S 1127, Department of Neurology, AP-HP, Pitié-Salpêtrière University Hospital, Sorbonne Universities, Pierre et Marie Curie University, Paris, France	Co-investigator
Serge Bracard	MD, PhD	Memory Resource and Research Centre of Nancy, CHU de Nancy, F-54000, Nancy, France	Co-investigator
Antoine Brangier	MD	Memory Resource and Research Centre of Angers, CHU d'Angers, F-49000, Angers	Co-investigator
Pierre-Yves Brillet	MD, PhD	Memory Clinic, Hôpital Avicenne, AP-HP, Hôpitaux Universitaires Paris-Seine-Saint-Denis, F-93009, Bobigny, France	Co-investigator
Laure Caillard	MD	Memory Resource and Research Centre of Paris Broca, AP-HP, Paris, France	Co-investigator
Fabienne Calvas	MD	Memory Resource and Research Centre of Toulouse, CHU de Toulouse, Hôpital Purpan, F-31000, Toulouse, France	Co-investigator
Agnès Camus	MD	Memory Resource and Research Centre of Dijon, CHU Dijon Bourgogne, Hôpital du Bocage, Hôpital de Champmaillot, F-21000, Dijon, France	Co-investigator
Vincent Camus	MD, PhD	Memory Resource and Research Centre of Center Region, CHRU de Tours, Hôpital Bretonneau, F-37000, Tours, France	Co-investigator
Sandrine Canaple	MD	Memory Resource and Research of Amiens, CHU Amiens Picardie, F-80000, Amiens, France	Co-investigator
Antoine Carpentier	MD, PhD	Memory Clinic, Hôpital Avicenne, AP-HP, Hôpitaux Universitaires Paris-Seine-Saint-Denis, F-93009, Bobigny, France	Co-investigator
Pascaline Cassagnaud	MD	Memory Resource and Research Centre of Lille, CHRU de Lille, Hôpital Roger Salengro, F-59000, Lille, France	Co-investigator
Françoise Cattin	MD	Memory Resource and Research Centre of Besançon, CHU de Besançon, Hôpital Jean Minjoz, Hôpital Saint-Jacques, F-25000, Besançon, France	Co-investigator
Ludivine Chamard	MD	Memory Resource and Research Centre of Besançon, CHU de Besançon, Hôpital Jean Minjoz, Hôpital Saint-Jacques, F-25000, Besançon, France	Co-investigator
Stéphane Chanalet	MD	Memory Resource and Research Centre of Nice, CHU de Nice, Hôpital Pasteur, F-06100, Nice, France	Co-investigator
Mathieu Chastan	MD	Memory Resource and Research Centre of Rouen, CLCC Henri Becquerel, Rouen, France	Co-investigator
Sophie Chauvelier	MD	Memory Resource and Research Centre of Paris Broca, AP-HP, Paris, France	Co-investigator
Valérie Chauvire	MD	Memory Resource and Research Centre of Angers, CHU d'Angers, F-49000, Angers	Co-investigator
Samia Cheriet	MD, PhD	Memory Resource and Research Centre of Toulouse, CHU de Toulouse, Hôpital Purpan, F-31000, Toulouse, France	Co-investigator
Anthony Clotagatide	MD	Memory Resource and Research Centre of Saint-Etienne, CHU de Saint-Etienne, Hôpital Nord, F-42000, Saint-Etienne, France	Co-investigator
Emmanuel Cognat	MD, PhD	Memory Resource and Research Centre of Paris Nord, AP-HP, Paris, France	Co-investigator
Lora Cohen	PhD	Memory Resource and Research Centre of Grenoble, CHU de Grenoble Alpes, Grenoble, France	Co-investigator
Jean-Marc Constans	MD, PhD	Memory Resource and Research of Amiens, CHU Amiens Picardie, F-80000, Amiens, France	Co-investigator
Marie-Hélène Coste	MD, PhD	Memory Resource and Research Centre of Lyon, Hospices Civils de Lyon, Hôpital des Charpennes, F-69000, Lyon, France	Co-investigator
Jean-Philippe Cottier	MD, PhD	Memory Resource and Research Centre of Center Region, CHRU de Tours, Hôpital Bretonneau, F-37000, Tours, France	Co-investigator
François Cotton	MD, PhD	Memory Resource and Research Centre of Lyon, Hospices Civils de Lyon, Hôpital des Charpennes, F-69000, Lyon, France	Co-investigator
Isabelle Couret	MD	Memory Resource and Research Centre of Montpellier, CHU de Montpellier, Hôpital Gui de Chauliac, F-34000, Montpellier, France	Co-investigator
Olivier-François Couturier	MD, PhD	Memory Resource and Research Centre of Angers, CHU d'Angers, F-49000, Angers	Co-investigator
Pascale Cowppli-Bony	MD, PhD	Memory Resource and Research Centre of Bordeaux, CHU de Bordeaux, Hôpital Pellegrin, F-33000, Bordeaux, France	Co-investigator
Véronique Cressot	MD	Memory Resource and Research Centre of Bordeaux, CHU de Bordeaux, Hôpital Xavier Arnozan, F-33000, Bordeaux, France	Co-investigator
Benjamin Crétin	MD	Memory Resource and Research Centre of Strasbourg, Hôpitaux Universitaires de Strasbourg, F-67000, Strasbourg, France	Co-investigator
Keren Danaila	MD	Memory Resource and Research Centre of Lyon, Hospices Civils de Lyon, Hôpital des Charpennes, F-69000, Lyon, France	Co-investigator

Jacques Darcourt	MD, PhD	Memory Resource and Research Centre of Nice, CLCC Antoine Lacassagne, Nice, France	Co-investigator
Jean-François Dartigues	MD, PhD	Memory Resource and Research Centre of Bordeaux, CHU de Bordeaux, Hôpital Pellegrin, F-33000, Bordeaux, France	Co-investigator
Ana-Maria Dascalita	MD, PhD	Memory Resource and Research Centre of Saint-Etienne, CHU de Saint-Etienne, Hôpital de la Charité, F-42000, Saint-Etienne, France	Co-investigator
Renaud David	MD, PhD	Memory Resource and Research Centre of Nice, CHU de Nice, Institut Claude Pompidou, F-06100, Nice, France	Co-investigator
Xavier De Petigny	MD	Memory Resource and Research Centre of Strasbourg, Hôpitaux Universitaires de Strasbourg, F-67000, Strasbourg, France	Co-investigator
Delphine De Verbizier-Lonjon	MD	Memory Resource and Research Centre of Montpellier, CHU de Montpellier, Hôpital Gui de Chauliac, F-34000, Montpellier, France	Co-investigator
Marielle Decousus	MD, PhD	Memory Resource and Research Centre of Saint-Etienne, CHU de Saint-Etienne, Hôpital Nord, F-42000, Saint-Etienne, France	Co-investigator
Isabelle Defouilloy	MD, PhD	Memory Resource and Research of Amiens, CHU Amiens Picardie, F-80000, Amiens, France	Co-investigator
Christine Delmaire	MD, PhD	Memory Resource and Research Centre of Lille, CHRU de Lille, Hôpital Roger Salengro, F-59000, Lille, France	Co-investigator
Julien Delrieu	MD	Memory Resource and Research Centre of Toulouse, CHU de Toulouse, Hôpital La Grave-Casselardit, F-31000, Toulouse, France	Co-investigator
Catherine Demuyinck	MD	Memory Resource and Research Centre of Strasbourg, Hôpitaux Universitaires de Strasbourg, F-67000, Strasbourg, France	Co-investigator
Vincent Deramecourt	MD, PhD	Memory Resource and Research Centre of Lille, CHRU de Lille, Hôpital Roger Salengro, F-59000, Lille, France	Co-investigator
Hervé Deramond	MD, PhD	Memory Resource and Research of Amiens, CHU Amiens Picardie, F-80000, Amiens, France	Co-investigator
Thomas Desmidt	MD, PhD	Memory Resource and Research Centre of Center Region, CHRU de Tours, Hôpital Bretonneau, F-37000, Tours, France	Co-investigator
Marie-Dominique Desruet	PharmD, PhD	Memory Resource and Research Centre of Grenoble, CHU de Grenoble Alpes, Grenoble, France	Co-investigator
Julien Detour	PharmD, PhD	Memory Resource and Research Centre of Strasbourg, Hôpitaux Universitaires de Strasbourg, F-67000, Strasbourg, France	Co-investigator
Agnès Devendeville	MD	Memory Resource and Research of Amiens, CHU Amiens Picardie, F-80000, Amiens, France	Co-investigator
Mira Didic	MD, PhD	Memory Resource and Research Centre of Marseille, CHU de Marseille, Hôpital La Timone, F-13000, Marseille, France	Co-investigator
Maritchu Doireau	MD	Memory Resource and Research Centre of Bordeaux, CHU de Bordeaux, Hôpital Pellegrin, F-33000, Bordeaux, France	Co-investigator
Antonio Dos Santos	MD	Institute of Memory and Alzheimer's Disease (IM2A), Brain and Spine Institute (ICM), UMR S 1127, Department of Neurology, AP-HP, Pitié-Salpêtrière University Hospital, Sorbonne Universities, Pierre et Marie Curie University, Paris, France	Co-investigator
Patrice Douillet	MD	Memory Resource and Research Centre of Montpellier, CHU de Montpellier, Hôpital Gui de Chauliac, F-34000, Montpellier, France	Co-investigator
Foucaud Du Boisgueheneuc	MD	Memory Resource and Research Centre of Poitiers, CHU de Poitiers, Hôpital de La Milétrie, F-86000, Poitiers, France	Co-investigator
Delphine Dubail	MD	Memory Resource and Research Centre of Paris Broca, AP-HP, Paris, France	Co-investigator
Laure Ducroq-Ducastaing	MD	Memory Resource and Research Centre of Brest, CHRU de Brest, F-29000, Brest, France	Co-investigator
Julien Dumurgier	MD, PhD	Memory Resource and Research Centre of Paris Nord, AP-HP, Paris, France	Co-investigator
Diane Dupuy	MD, PhD	Memory Resource and Research of Amiens, CHU Amiens Picardie, F-80000, Amiens, France	Co-investigator
Emmanuelle Duron	MD, PhD	Memory Resource and Research Centre of Paris Broca, AP-HP, Paris, France	Co-investigator
Inna Dygai-Cochet	MD, PhD	Memory Resource and Research Centre of Dijon, CLCC Georges François Leclerc, Dijon, France	Co-investigator
Véronique Eder	MD, PhD	Memory Clinic, Hôpital Avicenne, AP-HP, Hôpitaux Universitaires Paris-Seine-Saint-Denis, F-93009, Bobigny, France	Co-investigator
Stéphane Epelbaum	MD, PhD	Institute of Memory and Alzheimer's Disease (IM2A), Brain and Spine Institute (ICM), UMR S 1127, Department of Neurology, AP-HP, Pitié-Salpêtrière University Hospital, Sorbonne Universities, Pierre et Marie Curie University, Paris, France	Co-investigator
Frédérique Etcharry-Bouyx	MD, PhD	Memory Resource and Research Centre of Angers, CHU d'Angers, F-49000, Angers	Co-investigator

Daniel Fagret	MD, PhD	Memory Resource and Research Centre of Grenoble, CHU de Grenoble Alpes, Grenoble, France	Co-investigator
Catherine Faisant	MD	Memory Resource and Research Centre of Toulouse, CHU de Toulouse, Hôpital La Grave-Casselardit, F-31000, Toulouse, France	Co-investigator
Karim Farid	MD, PhD	Memory Resource and Research Centre of Paris Nord, AP-HP, Paris, France	Co-investigator
Denis Fédérico	MD	Memory Resource and Research Centre of Lyon, Hospices Civils de Lyon, Hôpital des Charpennes, F-69000, Lyon, France	Co-investigator
Olivier Felician	MD, PhD	Memory Resource and Research Centre of Marseille, CHU de Marseille, Hôpital La Timone, F-13000, Marseille, France	Co-investigator
Philippe Fernandez	MD, PhD	Memory Resource and Research Centre of Bordeaux, CHU de Bordeaux, Hôpital Pellegrin, F-33000, Bordeaux, France	Co-investigator
Pacôme Fosse	MD	Memory Resource and Research Centre of Angers, CHU d'Angers, F-49000, Angers	Co-investigator
Alexandra Foubert-Samier	MD, PhD	Memory Resource and Research Centre of Bordeaux, CHU de Bordeaux, Hôpital Pellegrin, F-33000, Bordeaux, France	Co-investigator
Isabelle Franck	MD	Memory Resource and Research Centre of Strasbourg, Hôpitaux Universitaires de Strasbourg, F-67000, Strasbourg, France	Co-investigator
Monique Galitzky	MD	Memory Resource and Research Centre of Toulouse, CHU de Toulouse, Hôpital Purpan, F-31000, Toulouse, France	Co-investigator
Céline Gallazzini-Crepin	MD	Memory Resource and Research Centre of Grenoble, CHU de Grenoble Alpes, Grenoble, France	Co-investigator
Radka Gantchev	MD	Memory Resource and Research Centre of Marseille, CHU de Marseille, Hôpital La Timone, F-13000, Marseille, France	Co-investigator
Laurence Garbarg-Chenon	MD	Memory Clinic, Hôpital Avicenne, AP-HP, Hôpitaux Universitaires Paris-Seine-Saint-Denis, F-93009, Bobigny, France	Co-investigator
Guillaume Gautier	MD, PhD	Memory Resource and Research Centre of Marseille, CHU de Marseille, Hôpital La Timone, F-13000, Marseille, France	Co-investigator
Emmanuel Gerardin	MD, PhD	Memory Resource and Research Centre of Rouen, Neuroradiology Department, Rouen University Hospital, F-76031, Rouen, France	Co-investigator
Claire Gervais	MD	Memory Resource and Research Centre of Nice, CHU de Nice, Institut Claude Pompidou, F-06100, Nice, France	Co-investigator
Jean-Claude Getenet	MD	Memory Resource and Research Centre of Saint-Etienne, CHU de Saint-Etienne, Hôpital Nord, F-42000, Saint-Etienne, France	Co-investigator
Nadine Girard	MD, PhD	Memory Resource and Research Centre of Marseille, CHU de Marseille, Hôpital La Timone, F-13000, Marseille, France	Co-investigator
Fabienne Giraud	MD	Memory Resource and Research Centre of Marseille, CHU de Marseille, Hôpital La Timone, F-13000, Marseille, France	Co-investigator
Chantal Girtanner	MD	Memory Resource and Research Centre of Saint-Etienne, CHU de Saint-Etienne, Hôpital de la Charité, F-42000, Saint-Etienne, France	Co-investigator
Valérie Gissot	MD	Memory Resource and Research Centre of Center Region, CHRU de Tours, Hôpital Bretonneau, F-37000, Tours, France	Co-investigator
Caroline Grangeon	PharmD	Memory Resource and Research Centre of Nice, CHU de Nice, Institut Claude Pompidou, F-06100, Nice, France	Co-investigator
Daniel Grucker	MD, PhD	Memory Resource and Research Centre of Strasbourg, Hôpitaux Universitaires de Strasbourg, F-67000, Strasbourg, France	Co-investigator
Eric Guedj	MD, PhD	Memory Resource and Research Centre of Marseille, CHU de Marseille, Hôpital La Timone, F-13000, Marseille, France	Co-investigator
Claude Gueriot	MD	Memory Resource and Research Centre of Marseille, CHU de Marseille, Hôpital La Timone, F-13000, Marseille, France	Co-investigator
Yves Guilhermet	MD	Memory Resource and Research Centre of Lyon, Hospices Civils de Lyon, Hôpital des Charpennes, F-69000, Lyon, France	Co-investigator
Rémy Guillevin	MD, PhD	Memory Resource and Research Centre of Poitiers, CHU de Poitiers, Hôpital de La Milétrie, F-86000, Poitiers, France	Co-investigator
Sophie Haffen	MD	Memory Resource and Research Centre of Besançon, CHU de Besançon, Hôpital Jean Minjoz, Hôpital Saint-Jacques, F-25000, Besançon, France	Co-investigator
Didier Hannequin	MD, PhD	Memory Resource and Research Centre of Rouen, Neurology Department, Rouen University Hospital, F-76031, Rouen, France	Co-investigator
Sandrine Harston	MD	Memory Resource and Research Centre of Bordeaux, CHU de Bordeaux, Hôpital Xavier Arnozan, F-33000, Bordeaux, France	Co-investigator
Anne Hitzel	MD, PhD	Memory Resource and Research Centre of Toulouse, CHU de Toulouse, Hôpital Purpan, F-31000, Toulouse, France	Co-investigator
Caroline Hommet	MD, PhD	Memory Resource and Research Centre of Center Region, CHRU de Tours, Hôpital Bretonneau, F-37000, Tours, France	Co-investigator
Claude Hossein-Foucher	MD, PhD	Memory Resource and Research Centre of Lille, CHRU de Lille, Hôpital Roger Salengro, F-59000, Lille, France	Co-investigator
Fabrice Hubele	MD	Memory Resource and Research Centre of Strasbourg, Hôpitaux Universitaires de Strasbourg, F-67000, Strasbourg, France	Co-investigator
Agnès	MD, PhD	Memory Resource and Research Centre of Dijon, CHU Dijon Bourgogne, Hôpital du Bocage,	Co-investigator

Jacquin-Piques		Hôpital de Champmaillot, F-21000, Dijon, France	
Betty Jean	MD	Memory Resource and Research Centre of Clermont-Ferrand, CHU de Clermont-Ferrand, F-63000, Clermont-Ferrand, France	Co-investigator
Joanne Jenn	MD, PhD	Memory Resource and Research Centre of Bordeaux, CHU de Bordeaux, Hôpital Xavier Arnozan, F-33000, Bordeaux, France	Co-investigator
Laure Joly	MD, PhD	Memory Resource and Research Centre of Nancy, CHU de Nancy, F-54000, Nancy, France	Co-investigator
Thérèse Jonveaux	MD	Memory Resource and Research Centre of Nancy, CHU de Nancy, F-54000, Nancy, France	Co-investigator
Adrien Julian	MD, PhD	Memory Resource and Research Centre of Poitiers, CHU de Poitiers, Hôpital de La Milétrie, F-86000, Poitiers, France	Co-investigator
Aurélie Kas	MD, PhD	Laboratoire d'Imagerie Biomédicale, Sorbonne Universités, UPMC Univ Paris 06, Inserm U1146, CNRS UMR 7371, France NeuroSpin, I2BM, Commissariat à l'Energie Atomique, Paris, France	Co-investigator
Anna Kearney-Schwartz	MD	Memory Resource and Research Centre of Nancy, CHU de Nancy, F-54000, Nancy, France	Co-investigator
Alice Keles	MD	Memory Resource and Research Centre of Nancy, CHU de Nancy, F-54000, Nancy, France	Co-investigator
Antony Kelly	MD	Memory Resource and Research Centre of Clermont-Ferrand, Centre de Lutte contre le Cancer, F-63000, Clermont-Ferrand, France	Co-investigator
Nathalie Keromnes	MD	Memory Resource and Research Centre of Brest, CHRU de Brest, F-29000, Brest, France	Co-investigator
Lejla Koric	MD	Memory Resource and Research Centre of Marseille, CHU de Marseille, Hôpital La Timone, F-13000, Marseille, France	Co-investigator
Alexandre Krainik	MD, PhD	Memory Resource and Research Centre of Grenoble, CHU de Grenoble Alpes, Grenoble, France	Co-investigator
Stéphane Kremer	MD	Memory Resource and Research Centre of Strasbourg, Hôpitaux Universitaires de Strasbourg, F-67000, Strasbourg, France	Co-investigator
Florian Labourée	MD	Memory Resource and Research Centre of Paris Broca, AP-HP, Paris, France	Co-investigator
Franck Lacoeuille	MD, PhD	Memory Resource and Research Centre of Angers, CHU d'Angers, F-49000, Angers	Co-investigator
Francoise Lala	MD	Memory Resource and Research Centre of Toulouse, CHU de Toulouse, Hôpital La Grave-Casselardit, F-31000, Toulouse, France	Co-investigator
Chantal Lamy	MD	Memory Resource and Research of Amiens, CHU Amiens Picardie, F-80000, Amiens, France	Co-investigator
Jean-Louis Laplanche	PharmD, PhD	Memory Resource and Research Centre of Paris Nord, AP-HP, Paris, France	Co-investigator
Cyrille Launay	MD, PhD	Memory Resource and Research Centre of Angers, CHU d'Angers, F-49000, Angers	Co-investigator
Stéphane Lehericy	MD, PhD	Institute of Memory and Alzheimer's Disease (IM2A), Centre for NeuroImaging Research (CENIR), Brain and Spine Institute (ICM), UMR S 1127, Department of Neurology, AP-HP, Pitié-Salpêtrière University Hospital, Sorbonne Universities, Pierre et Marie Curie University, Paris, France	Co-investigator
Sylvain Lehmann	MD, PhD	Memory Resource and Research Centre of Montpellier, CHU de Montpellier, Hôpital Gui de Chauliac, F-34000, Montpellier, France	Co-investigator
Hermine Lenoir	MD, PhD	Memory Resource and Research Centre of Paris Broca, AP-HP, Paris, France	Co-investigator
Marcel Levy	MD, PhD	Institute of Memory and Alzheimer's Disease (IM2A), Brain and Spine Institute (ICM), UMR S 1127, Department of Neurology, AP-HP, Pitié-Salpêtrière University Hospital, Sorbonne Universities, Pierre et Marie Curie University, Paris, France	Co-investigator
Stéphanie Libercier	MD, PhD	Memory Resource and Research Centre of Colmar, Hôpitaux Civils de Colmar, F-68000, Colmar, France	Co-investigator
Marie-Anne Mackowiak-Cordoliani	MD	Memory Resource and Research Centre of Lille, CHRU de Lille, Hôpital Roger Salengro, F-59000, Lille, France	Co-investigator
Eloi Magnin	MD	Memory Resource and Research Centre of Besançon, CHU de Besançon, Hôpital Jean Minjoz, Hôpital Saint-Jacques, F-25000, Besançon, France	Co-investigator
Zaza Makaroff	MD	Memory Resource and Research Centre of Lyon, Hospices Civils de Lyon, Hôpital des Charpennes, F-69000, Lyon, France	Co-investigator
Athina Marantidou	MD	Memory Clinic, Hôpital Avicenne, AP-HP, Hôpitaux Universitaires Paris-Seine-Saint-Denis, F-93009, Bobigny, France	Co-investigator
Isabelle Marcet	MD	Memory Resource and Research Centre of Bordeaux, CHU de Bordeaux, Hôpital Pellegrin, F-33000, Bordeaux, France	Co-investigator
Cécilia Marelli	MD, PhD	Memory Resource and Research Centre of Montpellier, CHU de Montpellier, Hôpital Gui de Chauliac, F-34000, Montpellier, France	Co-investigator
Sophie Marilier	MD	Memory Resource and Research Centre of Dijon, CHU Dijon Bourgogne, Hôpital du Bocage, Hôpital de Champmaillot, F-21000, Dijon, France	Co-investigator
Idalie Martin	MD	Memory Resource and Research Centre of Lyon, Hospices Civils de Lyon, Hôpital des Charpennes, F-69000, Lyon, France	Co-investigator
Olivier Martinaud	MD, PhD	Memory Resource and Research Centre of Rouen, Neurology Department, Rouen University Hospital, F-76031, Rouen, France	Co-investigator
Catherine Martin-	MD	Memory Resource and Research Centre of Strasbourg, Hôpitaux Universitaires de Strasbourg, F-67000, Strasbourg, France	Co-investigator

Hunyadi			
Aïcha Medjoul	MD	Memory Clinic, Hôpital Avicenne, AP-HP, Hôpitaux Universitaires Paris-Seine-Saint-Denis, F-93009, Bobigny, France	Co-investigator
Isabelle Merlet	MD	Memory Resource and Research Centre of Poitiers, CHU de Poitiers, Hôpital de La Milétrie, F-86000, Poitiers, France	Co-investigator
Danielle Mestas	MD	Memory Resource and Research Centre of Clermont-Ferrand, CHU de Clermont-Ferrand, F-63000, Clermont-Ferrand, France	Co-investigator
Marc-Etienne Meyer	MD, PhD	Memory Resource and Research of Amiens, CHU Amiens Picardie, F-80000, Amiens, France	Co-investigator
Jean-Marc Michel	MD	Memory Resource and Research Centre of Colmar, Hôpitaux Civils de Colmar, F-68000, Colmar, France	Co-investigator
Agnès Michon	MD	Institute of Memory and Alzheimer's Disease (IM2A), Brain and Spine Institute (ICM), UMR S 1127, Department of Neurology, AP-HP, Pitié-Salpêtrière University Hospital, Sorbonne Universities, Pierre et Marie Curie University, Paris, France	Co-investigator
Isabelle Migeon-Duballet	MD	Memory Resource and Research Centre of Poitiers, CHU de Poitiers, Hôpital de La Milétrie, F-86000, Poitiers, France	Co-investigator
Sophie Mohr	MD	Memory Resource and Research Centre of Dijon, CHU Dijon Bourgogne, Hôpital du Bocage, F-21000, Dijon, France	Co-investigator
Karl Mondon	MD, PhD	Memory Resource and Research Centre of Center Region, CHRU de Tours, Hôpital Bretonneau, F-37000, Tours, France	Co-investigator
Clément Morgat	PharmD, PhD	Memory Resource and Research Centre of Bordeaux, CHU de Bordeaux, Hôpital Pellegrin, F-33000, Bordeaux, France	Co-investigator
Véronique Moullart	MD	Memory Resource and Research of Amiens, CHU Amiens Picardie, F-80000, Amiens, France	Co-investigator
Christian Moussard	MD	Memory Resource and Research Centre of Besançon, CHU de Besançon, Hôpital Jean Minjoz, Hôpital Saint-Jacques, F-25000, Besançon, France	Co-investigator
Aurélie Mouton	MD, PhD	Memory Resource and Research Centre of Nice, CHU de Nice, Institut Claude Pompidou, F-06100, Nice, France	Co-investigator
Izzie Jacques Namer	MD, PhD	Memory Resource and Research Centre of Strasbourg, Hôpitaux Universitaires de Strasbourg, F-67000, Strasbourg, France	Co-investigator
Georges Niewiadomski	MD, PhD	Memory Resource and Research Centre of Nice, CHU de Nice, Institut Claude Pompidou, F-06100, Nice, France	Co-investigator
Guillaume Nivaggioni	MD	Memory Resource and Research Centre of Nice, CHU de Nice, Institut Claude Pompidou, F-06100, Nice, France	Co-investigator
Marie Noblet	MD, PhD	Memory Resource and Research Centre of Strasbourg, Hôpitaux Universitaires de Strasbourg, F-67000, Strasbourg, France	Co-investigator
Michel Nonent	MD, PhD	Memory Resource and Research Centre of Brest, CHRU de Brest, F-29000, Brest, France	Co-investigator
Fati Nourhashemi	MD, PhD	Memory Resource and Research Centre of Toulouse, CHU de Toulouse, Hôpital La Grave-Casselardit, F-31000, Toulouse, France	Co-investigator
Hélène Oesterle	MD	Memory Resource and Research Centre of Colmar, Hôpitaux Civils de Colmar, F-68000, Colmar, France	Co-investigator
Galdric Orvoen	MD	Memory Resource and Research Centre of Paris Broca, AP-HP, Paris, France	Co-investigator
Pierre Jean Ousset	MD, PhD	Memory Resource and Research Centre of Toulouse, CHU de Toulouse, Hôpital La Grave-Casselardit, F-31000, Toulouse, France	Co-investigator
Amandine Pallardy	MD	Memory Resource and Research Centre of Nantes, CHU de Nantes, F-44000, Nantes, France	Co-investigator
Claire Paquet	MD, PhD	Memory Resource and Research Centre of Paris Nord, AP-HP, Paris, France	Co-investigator
Pierre-Yves Pare	MD, PhD	Memory Resource and Research Centre of Angers, CHU d'Angers, F-49000, Angers	Co-investigator
Anne Pasco	MD, PhD	Memory Resource and Research Centre of Angers, CHU d'Angers, F-49000, Angers	Co-investigator
Pierre Payoux	MD, PhD	Memory Resource and Research Centre of Toulouse, CHU de Toulouse, Hôpital Purpan, F-31000, Toulouse, France	Co-investigator
Cécile Pays	MD, PhD	Memory Resource and Research Centre of Montpellier, CHU de Montpellier, Hôpital Gui de Chauliac, F-34000, Montpellier, France	Co-investigator
Isabelle Pellegrin	MD, PhD	Biological Research Centre, CHU de Bordeaux, F-33000, Bordeaux, France	Co-investigator
Rémy Perdrisot	MD, PhD	Memory Resource and Research Centre of Poitiers, CHU de Poitiers, Hôpital de La Milétrie, F-86000, Poitiers, France	Co-investigator
Bertille Perin	MD, PhD	Memory Resource and Research of Amiens, CHU Amiens Picardie, F-80000, Amiens, France	Co-investigator
Christine Perret-Guillaume	MD, PhD	Memory Resource and Research Centre of Nancy, CHU de Nancy, F-54000, Nancy, France	Co-investigator
Grégory Petyt	MD	Memory Resource and Research Centre of Lille, CHRU de Lille, Hôpital Roger Salengro, F-59000, Lille, France	Co-investigator

Nathalie Philippi	MD, PhD	Memory Resource and Research Centre of Strasbourg, Hôpitaux Universitaires de Strasbourg, F-67000, Strasbourg, France	Co-investigator
Geneviève Pinganaud	MD	Memory Resource and Research Centre of Bordeaux, CHU de Bordeaux, Hôpital Xavier Arnozan, F-33000, Bordeaux, France	Co-investigator
Vincent Planche	MD, PhD	Memory Resource and Research Centre of Bordeaux, CHU de Bordeaux, Hôpital Pellegrin, F-33000, Bordeaux, France	Co-investigator
Matthieu Plichart	MD	Memory Resource and Research Centre of Paris Broca, AP-HP, Paris, France	Co-investigator
Gabriel Pop	MD, PhD	Memory Clinic, Hôpital Avicenne, AP-HP, Hôpitaux Universitaires Paris-Seine-Saint-Denis, F-93009, Bobigny, France	Co-investigator
Michèle Puel	MD	Memory Resource and Research Centre of Toulouse, CHU de Toulouse, Hôpital Purpan, F-31000, Toulouse, France	Co-investigator
Mathieu Queneau	MD, PhD	Memory Resource and Research Centre of Paris Nord, Centre Cardiologique du Nord, Paris, France	Co-investigator
Solène Querellou	MD	Memory Resource and Research Centre of Brest, CHRU de Brest, F-29000, Brest, France	Co-investigator
Muriel Quillard-Muraine	MD, PhD	Memory Resource and Research Centre of Rouen, Neurology Department, Rouen University Hospital, F-76031, Rouen, France	Co-investigator
Valérie Quipourt	MD, PhD	Memory Resource and Research Centre of Dijon, CHU Dijon Bourgogne, Hôpital du Bocage, Hôpital de Champmaillot, F-21000, Dijon, France	Co-investigator
Chloé Rachez	MD, PhD	Memory Resource and Research Centre of Clermont-Ferrand, CHU de Clermont-Ferrand, F-63000, Clermont-Ferrand, France	Co-investigator
Micheline Razzouk-Cadet	MD	Memory Resource and Research Centre of Nice, CHU de Nice, Institut Claude Pompidou, F-06100, Nice, France	Co-investigator
Anne-Sophie Rigaud	MD, PhD	Memory Resource and Research Centre of Paris Broca, AP-HP, Paris, France	Co-investigator
Hélène Robin-Ismer	MD	Memory Resource and Research Centre of Strasbourg, Hôpitaux Universitaires de Strasbourg, F-67000, Strasbourg, France	Co-investigator
Mathieu Rodallec	MD, PhD	Memory Resource and Research Centre of Paris Nord, Centre Cardiologique du Nord, Paris, France	Co-investigator
Yves Rolland	MD, PhD	Memory Resource and Research Centre of Toulouse, CHU de Toulouse, Hôpital La Grave-Casselardit, F-31000, Toulouse, France	Co-investigator
Adeline Rollin-Sillaire	MD, PhD	Memory Resource and Research Centre of Lille, CHRU de Lille, Hôpital Roger Salengro, F-59000, Lille, France	Co-investigator
Olivier Rouaud	MD	Memory Resource and Research Centre of Dijon, CHU Dijon Bourgogne, Hôpital du Bocage, Hôpital de Champmaillot, F-21000, Dijon, France	Co-investigator
Caroline Roubaud	MD, PhD	Memory Resource and Research Centre of Lyon, Hospices Civils de Lyon, Hôpital des Charpennes, F-69000, Lyon, France	Co-investigator
Isabelle Rouch	MD, PhD	Memory Resource and Research Centre of Lyon, Hospices Civils de Lyon, Hôpital des Charpennes, F-69000, Lyon, France	Co-investigator
Julie Roux	MD, PhD	Memory Resource and Research Centre of Grenoble, CHU de Grenoble Alpes, Grenoble, France	Co-investigator
Guillaume Sacco	MD, PhD	Memory Resource and Research Centre of Nice, CHU de Nice, Institut Claude Pompidou, F-06100, Nice, France	Co-investigator
Pierre-Yves Salaun	MD	Memory Resource and Research Centre of Brest, CHRU de Brest, F-29000, Brest, France	Co-investigator
François Salmon	MD, PhD	Memory Resource and Research Centre of Poitiers, CHU de Poitiers, Hôpital de La Milétrie, F-86000, Poitiers, France	Co-investigator
Alicia Sanchez	MD	Memory Resource and Research Centre of Saint-Etienne, CHU de Saint-Etienne, Hôpital Nord, F-42000, Saint-Etienne, France	Co-investigator
Maria-Joao Santiago-Ribeiro	MD, PhD	Memory Resource and Research Centre of Center Region, CHRU de Tours, Hôpital Bretonneau, F-37000, Tours, France	Co-investigator
Alain Sarciron	MD	Memory Resource and Research Centre of Lyon, Hospices Civils de Lyon, Hôpital des Charpennes, F-69000, Lyon, France	Co-investigator
Nathalie Sastre-Hengan	MD	Memory Resource and Research Centre of Toulouse, CHU de Toulouse, Hôpital La Grave-Casselardit, F-31000, Toulouse, France	Co-investigator
Mathilde Sauvée	MD, PhD	Memory Resource and Research Centre of Grenoble, CHU de Grenoble Alpes, Grenoble, France	Co-investigator
Christian Scheiber	MD, PhD	Memory Resource and Research Centre of Lyon, Hospices Civils de Lyon, Hôpital des Charpennes, F-69000, Lyon, France	Co-investigator
Anne-Marie Schneider	MD, PhD	Memory Resource and Research Centre of Strasbourg, Hôpitaux Universitaires de Strasbourg, F-67000, Strasbourg, France	Co-investigator
Franck Semah	MD, PhD	Memory Resource and Research Centre of Lille, CHRU de Lille, Hôpital Roger Salengro, F-59000, Lille, France	Co-investigator
Amélie Serra	MD	Memory Resource and Research Centre of Grenoble, CHU de Grenoble Alpes, Grenoble, France	Co-investigator
Marie-Laure Seux	MD	Memory Resource and Research Centre of Paris Broca, AP-HP, Paris, France	Co-investigator

Hélène Sordet-Guépet	MD	Memory Resource and Research Centre of Dijon, CHU Dijon Bourgogne, Hôpital du Bocage, Hôpital de Champmaillot, F-21000, Dijon, France	Co-investigator
Maria Eugenia Soto	MD	Memory Resource and Research Centre of Toulouse, CHU de Toulouse, Hôpital La Grave-Casselardit, F-31000, Toulouse, France	Co-investigator
Mathieu Tafani	MD	Memory Resource and Research Centre of Toulouse, CHU de Toulouse, Hôpital Purpan, F-31000, Toulouse, France	Co-investigator
Jean-Yves Tanguy	MD, PhD	Memory Resource and Research Centre of Angers, CHU d'Angers, F-49000, Angers	Co-investigator
Michael Taroux	MD, PhD	Memory Resource and Research Centre of Dijon, CHU Dijon Bourgogne, Hôpital du Bocage, Hôpital de Champmaillot, F-21000, Dijon, France	Co-investigator
Marc Teichmann	MD, PhD	Institute of Memory and Alzheimer's Disease (IM2A), Brain and Spine Institute (ICM), UMR S 1127, Department of Neurology, AP-HP, Pitié-Salpêtrière University Hospital, Sorbonne Universities, Pierre et Marie Curie University, Paris, France	Co-investigator
Catherine Terrat	MD, PhD	Memory Resource and Research Centre of Saint-Etienne, CHU de Saint-Etienne, Hôpital de la Charité, F-42000, Saint-Etienne, France	Co-investigator
Jamila Thabet	MD	Memory Clinic, Hôpital Avicenne, AP-HP, Hôpitaux Universitaires Paris-Seine-Saint-Denis, F-93009, Bobigny, France	Co-investigator
Claire Thalamas	MD	Memory Resource and Research Centre of Toulouse, CHU de Toulouse, Hôpital Purpan, F-31000, Toulouse, France	Co-investigator
Catherine Thomas-Anterion	MD, PhD	Memory Resource and Research Centre of Saint-Etienne, CHU de Saint-Etienne, Hôpital Nord, F-42000, Saint-Etienne, France	Co-investigator
Anne-Cécile Troussière	MD	Memory Resource and Research Centre of Lille, CHRU de Lille, Hôpital Roger Salengro, F-59000, Lille, France	Co-investigator
Renata Ursu	MD	Memory Clinic, Hôpital Avicenne, AP-HP, Hôpitaux Universitaires Paris-Seine-Saint-Denis, F-93009, Bobigny, France	Co-investigator
Pierre Vera	MD, PhD	Memory Resource and Research Centre of Rouen, CLCC Henri Becquerel, Rouen, France	Co-investigator
Martine Vercelletto	MD	Memory Resource and Research Centre of Nantes, CHU de Nantes, F-44000, Nantes, France	Co-investigator
Olivier Vercruysse	MD	Memory Resource and Research Centre of Lille, CHRU de Lille, Hôpital Roger Salengro, F-59000, Lille, France	Co-investigator
Antoine Verger	MD, PhD	Memory Resource and Research Centre of Nancy, CHU de Nancy, F-54000, Nancy, France	Co-investigator
Philippe Viau	MD	Memory Resource and Research Centre of Nice, CHU de Nice, Institut Claude Pompidou, F-06100, Nice, France	Co-investigator
Marie-Neige Videau	MD	Memory Resource and Research Centre of Bordeaux, CHU de Bordeaux, Hôpital Xavier Arnozan, F-33000, Bordeaux, France	Co-investigator
Thierry Voisin	MD	Memory Resource and Research Centre of Toulouse, CHU de Toulouse, Hôpital La Grave-Casselardit, F-31000, Toulouse, France	Co-investigator
Nathalie Wagemann	MD, PhD	Memory Resource and Research Centre of Nantes, CHU de Nantes, F-44000, Nantes, France	Co-investigator
Aziza Waissi-Sediq	MD	Memory Resource and Research Centre of Lyon, Hospices Civils de Lyon, Hôpital des Charpennes, F-69000, Lyon, France	Co-investigator
Jing Xie	MD, PhD	Memory Resource and Research Centre of Lyon, Hospices Civils de Lyon, Hôpital des Charpennes, F-69000, Lyon, France	Co-investigator
Nathanaëlle Yeni	MD	Laboratoire d'Imagerie Biomédicale, Sorbonne Universités, UPMC Univ Paris 06, Inserm U1146, CNRS UMR 7371, France NeuroSpin, I2BM, Commissariat à l'Energie Atomique, Paris, France	Co-investigator
Michel Zanca	MD, PhD	Memory Resource and Research Centre of Montpellier, CHU de Montpellier, Hôpital Gui de Chauliac, F-34000, Montpellier, France	Co-investigator
Jean Zinszner	MD, PhD	Memory Clinic, Hôpital Avicenne, AP-HP, Hôpitaux Universitaires Paris-Seine-Saint-Denis, F-93009, Bobigny, France	Co-investigator
